# 622. Evaluation of Vascular Access Device Selection in Patients Discharged on Outpatient Parenteral Antimicrobial Therapy

**DOI:** 10.1093/ofid/ofab466.820

**Published:** 2021-12-04

**Authors:** Jessica Hu, Lauren Dutcher, Vasilios Athans, Shawn Binkley, Justin Harris, Sonal Patel, Stephen Saw, Tiffany Lee

**Affiliations:** Hospital of the University of Pennsylvania, Philadelphia, Pennsylvania

## Abstract

**Background:**

Selection of a vascular access device (VAD) is an important consideration for patients receiving outpatient parenteral antimicrobial therapy (OPAT). Midline catheters (MC) and peripherally inserted central catheters (PICC) are the most commonly placed VADs, with the former recommended by national guidelines to be used for durations no longer than two weeks. These recommendations, however, are based on limited data from heterogeneous populations. As such, we aim to further characterize VAD-associated complications specifically in patients receiving antimicrobials.

**Methods:**

We conducted a retrospective cohort study that included adult patients discharged on OPAT with a newly inserted MC or PICC between January 2020 and August 2020. Patients with non-OPAT VAD indications were excluded. The primary outcome was the incidence of VAD-associated complications, which was further assessed by type and severity. The secondary outcome was time to complication. Multivariable Poisson regression was used to assess the association between VAD type and incidence of VAD-associated complications.

**Results:**

A total of 190 encounters from 181 patients were included for analysis. Baseline demographics are detailed in Table 1. Despite a higher number of complications in the PICC group, rates per 1000 VAD days were not significantly different between VAD types (Table 2). Median time to first complication was 17 days in the overall cohort. Multivariable regression analysis showed those with a dermatologic history had a four-fold increased risk for VAD-associated complications (Table 3). VAD type was not independently associated with the risk of developing a complication.

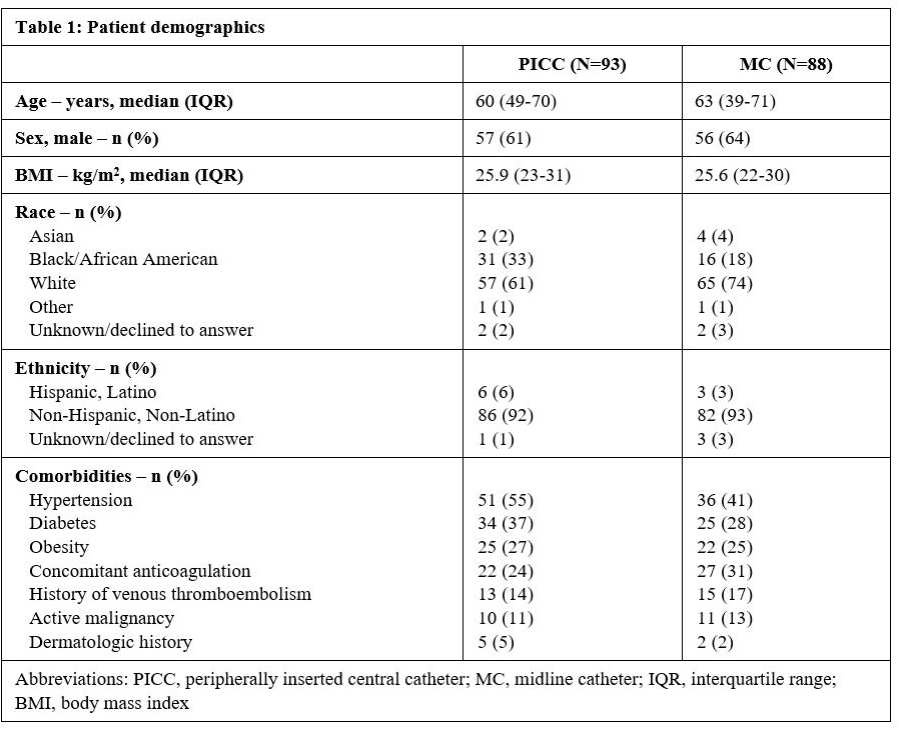

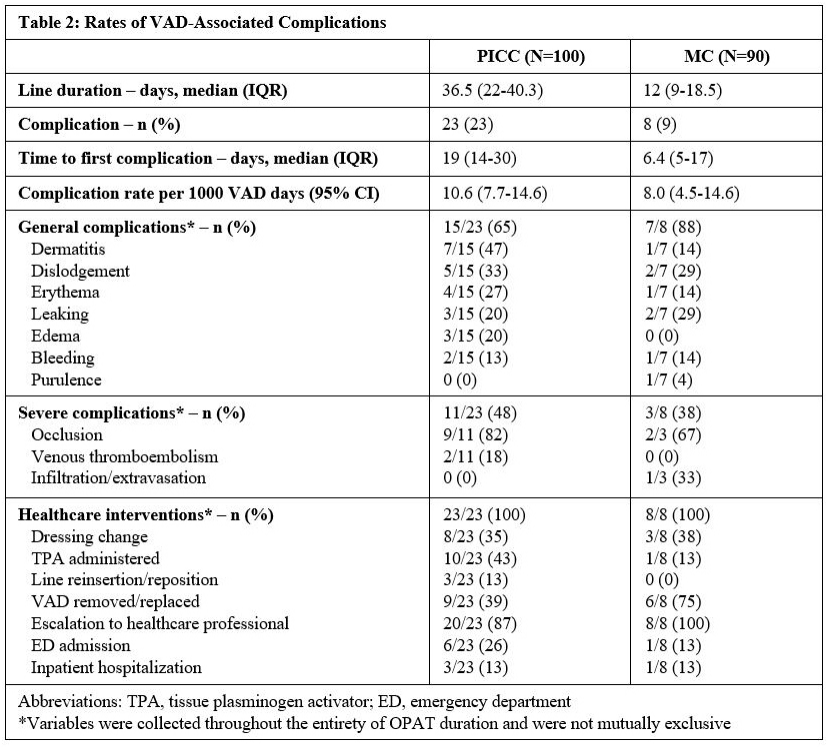

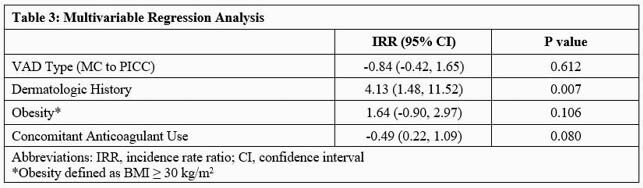

**Conclusion:**

Our results suggest that the development of VAD-associated complications was strongly associated with patients’ dermatologic history. To our knowledge, dermatologic history has not been previously identified as a risk factor for VAD-associated complications. Thorough assessment of patient-specific risk factors can inform optimal VAD selection for patients discharged on OPAT. Further studies are needed to assess the safety of MC for extended OPAT use.

**Disclosures:**

**All Authors**: No reported disclosures

